# Regulating the modulus of a chiral liquid crystal polymer network by light†
†Electronic supplementary information (ESI) available. See DOI: 10.1039/c6sm00114a



**DOI:** 10.1039/c6sm00114a

**Published:** 2016-02-15

**Authors:** Kamlesh Kumar, Albertus P. H. J. Schenning, Dirk J. Broer, Danqing Liu

**Affiliations:** a Laboratory of Functional Organic Materials & Devices (SFD) , Department of Chemical Engineering & Chemistry , Eindhoven University of Technology , De Rondom 70 , 5612 AP , Eindhoven , The Netherlands . Email: d.liu1@tue.nl; b Institute for Complex Molecular Systems (ICMS) , Eindhoven University of Technology , De Rondom 70 , 5612 AP , Eindhoven , The Netherlands

## Abstract

We report a novel way to modulate the elastic modulus of azobenzene containing liquid crystal networks (LCNs) by exposure to light. The elastic modulus can cycle between different levels by controlling the illumination conditions. Exposing the polymer network to UV light near the *trans* absorption band of azobenzene gives a small reduction of the glass transition temperature thereby lowering the modulus. The addition of blue light addressing the *cis* absorption band surprisingly amplifies this effect. The continuous oscillatory effects of the *trans*-to-*cis* isomerization of the azobenzene overrule the overall net *cis* conversion. The influence on the chain dynamics of the network is demonstrated by dynamic mechanical thermal analysis which shows a large shift of the glass transition temperature and a modulus decrease by more than two orders of magnitude. The initial high modulus and the glassy state are recovered within a minute in the dark by switching off the light sources, despite the observation that azobenzene is still predominantly in its *cis* state. Based on these new findings, we are able to create a shape memory polymer LCN film at room temperature using light.

## Abstract 2

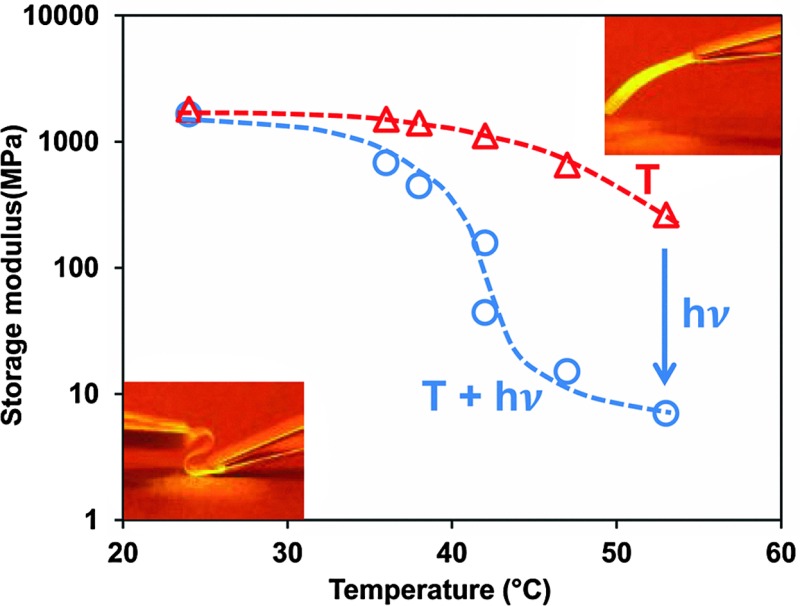
We provide a methodology to change the state of matter from hard and glassy to soft and rubbery by light.

## Introduction

Molecules that respond to external stimuli by changing their orientation, geometrical shape or breaking/forming chemical bonds are of interest for, among others, textiles, medical and aerospace industries. Often employed stimuli are light,^1,2^ chemicals^3,4^ and electrical^5,6^ or magnetic fields.^7–10^ Light is one of the preferred triggers and for this purpose azobenzene as the photoresponsive molecule has been widely studied.^11–15^ This photoresponsivity is characterized by a reversible transformation between *trans* and *cis* isomers. Upon illumination with UV light, preferably in its absorption band at 365 nm, it undergoes a transition from its ground *trans* state to the thermally more unstable *cis* state. The reverse reaction to the *trans* state may occur thermally or by illumination with white light where the absorption band of the *cis* state at around 450 nm is addressed. Embedding this molecule in a matrix of an ordered molecular system, such as a liquid crystal polymer network, introduces molecular cooperativity and directionality. Most often, the collective effect of the azobenzene isomerization process is demonstrated in macroscopic geometric deformations. A number of research groups have shown morphing behaviours, ranging from a free standing film that bends,^16,17^ curls^18,19^ or even transforms into more complex origami deformations^20^ to confined coatings that undergo from flat to the pre-designed surface topographical changes.^21^ The question that remains unanswered is how the often glassy polymer networks can undergo such large geometrical changes without fatigue on the time scale of the experiments. To gain more insight into the processes we study in this work how the oscillatory shape changes of azobenzene units influence the mechanical properties of the polymer matrix in which it is embedded.

Recently, several articles have been reported on the decrease of the elastic modulus of azobenzene containing linear polymers during light illumination.^22–26^ Terminologies such as ‘photo-fluidization’, ‘photo-softening’, and ‘photo-plasticization’ are used to describe this phenomenon. It is well characterized by a range of methodologies including nano-indentation, infrared spectroscopy, atomic force microscopy, and rheological measurements. These studies are mainly based on linear polymers where the chains can flow easily. Therefore, mass displacement or materials transport has been demonstrated in some cases. Mass transport can be prevented by choosing the crosslinked polymer networks. In crosslinked networks mass transport over larger distances, in relation to molecular length scales, is not possible. Instead the eventual small displacements and molecular reorganizations will be reversible as soon as the light source is taken away. It was demonstrated by the Air Force Research Laboratory that in densely crosslinked liquid crystal networks modified with 20% azobenzene the modulus can be reduced by one to two orders of magnitude upon exposure to 442 nm polarized light.^27–30^ We build further on this work by minimizing the azobenzene concentration to 2 wt% to further reduce eventual temperature effects. Subsequently we explore the dual-wavelength exposure to enhance the oscillation dynamics of azobenzene by simultaneously addressing the *trans* and the *cis* state of the azobenzene in an adjustable ratio.^31^ We show that under this illumination the elastic modulus of the polymer film can be further reduced by three orders of magnitude in combination with an optimized crosslink density. The results might lead to new applications. An example that was demonstrated before^27,28^ is the use of photo-plasticization for shape memory purposes.

## Results and discussion

**Scheme 1:**
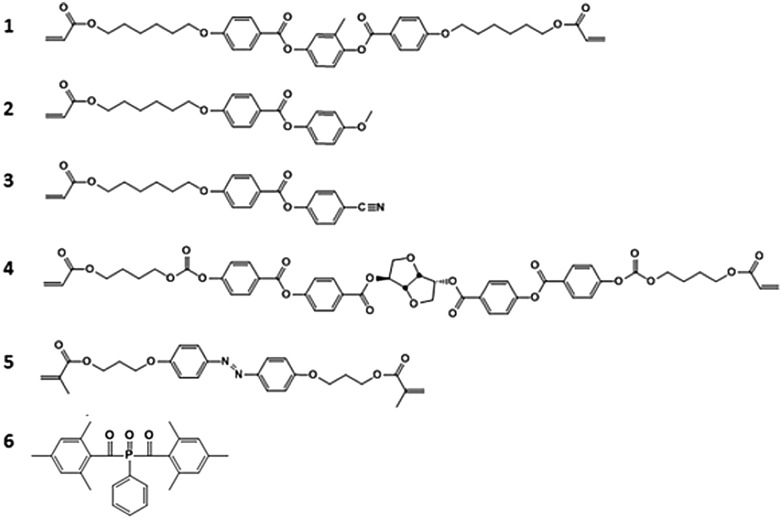
Chemicals used to fabricate the nematic cholesteric liquid crystalline network.

**Fig. 1:**
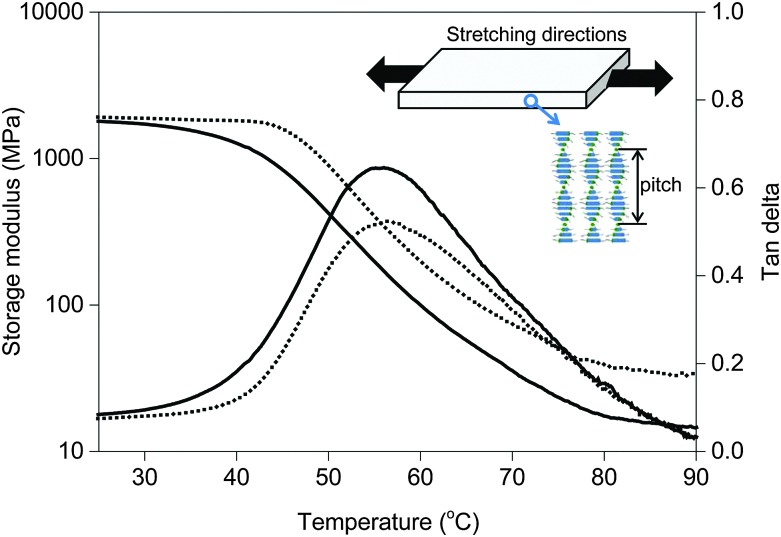
Stretching direction with respect to liquid crystal alignment in polymerized films (inset). Storage modulus and tan delta as a function of temperature of two tested chiral-nematic polymer networks (bottom). The solid line relates to the LCN with low crosslink density and the dashed line is the LCN with high crosslink density.

For this study, we create a composite of a blend of liquid crystal acrylates and some additives as shown in Scheme 1 (Experimental materials and methods). The composition is based on previously reported chiral-nematic LCN coatings that show light responsive topographical changes.^31^ Monomers 1–3 exhibit a nematic phase in which diacrylate 1 is a crosslinker that is used to balance the mechanical properties of the polymer. Chiral component 4 is added to induce the chiral-nematic phase. In the chiral-nematic phase the rodlike monomeric units are ordered on average in the plane of the film while their orientational axes describe a helix with its axis perpendicular to the film surface. The inset in Fig. 1 shows a schematic representation of the molecular organization. The pitch of the helix is around 400 nm. Monomer 5 has the azobenzene unit which provides the photo-mechanical response to the LCN. Previously we have found that a small amount as low as 2 wt% is sufficient to produce significant deformation effects.^32^ Using low concentration of azobenzene not only reduces heating by light absorption but also limits the attenuation of light intensity along the film thickness during actuation. Photoinitiator 6 is chosen as it can be activated by wavelengths >400 nm preventing the premature conversion of the azobenzene compound at an early stage during the photopolymerization process. Films were produced as discussed in the Experimental section while keeping the azobenzene in its *trans* state during the photopolymerization process. Two LCNs with different crosslink densities were prepared by changing the amount of diacrylate.^33^ For the higher crosslinked composition, the concentration of the diacrylate is increased from 22 to 32 wt% at the expense of the concentration of the monoacrylates.

The mechanical properties of the free standing liquid crystal polymer films were measured by dynamic mechanical thermal analysis (DMTA) (Fig. 1). The storage modulus values of the low and high-crosslinked samples are around 1.8 GPa and 2 GPa at room temperature and the glass transition temperatures analysed from the peak maximum of tan delta are about 55 °C and 57 °C, respectively. The tan delta curve of the high-crosslinked sample is broader to the high temperature side, possibly pointing to some inhomogeneities in the polymer network. The maximum derivative is therefore shifted at around 10 °C, somewhat more than the shift of tan delta maximum. The main difference induced by the high crosslink density is the increased level of the rubbery plateau, in accordance with the expectation.

**Fig. 2:**
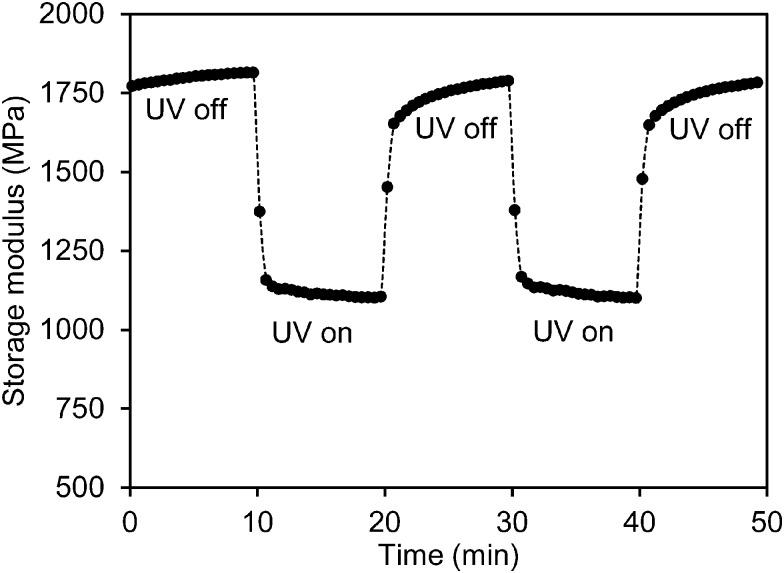
Modulus modulation of the azobenzene modified liquid crystal polymer network upon exposure to a 365 nm LED source with an intensity of 200 mW cm^–2^.

To study the effect of UV light on the mechanical properties, the storage modulus of the low-crosslinked film was first recorded during exposure to a UV LED light source (365 nm) with an intensity of 200 mW cm^–2^. During the measurement the LED lamp was switched on and off several times. Fig. 2 shows that as soon as the exposure starts the modulus responds immediately. It decreases sharply within one minute from 1.8 GPa to 1.1 GPa after which it stabilizes. Upon removal of the UV source the network relaxes within 1 minute back to its initial high modulus. This is a remarkable observation as the chemical relaxation of the *cis* azobenzene to its *trans* form is a process of several hours.^31^ This effect can be repeated several times (Fig. 2 shows two cycles). Similar observations were done during shear experiments of liquid crystal side-chain polymers with a high load of azobenzene moieties.^26^ In that case the experiments were done above the glass transition temperature and the explanation was found in the transition from the liquid crystalline to the isotropic phase as induced by the formation of the not-shape complying *cis*-azobenzene. In the experiment shown in Fig. 2 the liquid crystal order is maintained during the experiments, nevertheless still the modulus is reduced. It should also be noticed that the stress experiments were performed perpendicular to the helix axes of the chiral-nematic axes in a direction that the modulus is not sensitive for the order parameter as demonstrated in a comparison between the modulus of a chiral LC network and an isotropic network of the same composition.^34^

During UV exposure the temperature was measured to be 32 °C (Fig. S1 (ESI†)). The temperature rise does not explain the modulus reduction as can be concluded from Fig. 1 which shows only a marginal decrease of the modulus at this temperature. Upon illumination with UV light, azobenzene undergoes a photoisomerization from its rod shape *trans* to the bent *cis* state. The conversion reduces the order of the liquid crystal network and is reported to cause contraction along the molecular orientation and expansion to the perpendicular directions.^35^ In the case of the chiral-nematic order the net effect would be some shrinkage in the plane of the film and expansion perpendicular to that. The out of plane reorientation of the rod shaped molecular units upon reduction of the order parameter might explain some decrease of modulus but by far not enough to explain the reduction observed in Fig. 2. Ruling out the effects of temperature and director reorientation, plasticization of the polymer network chains under oscillatory stresses induced by the azobenzene molecules seems to be the most logical explanation. It also explains why the relaxation of the network in the dark to its initial high modulus is much faster than the relaxation of the azobenzene to its *trans* form. This postulation will be studied further by enhancing the oscillatory actions of the azobenzene crosslinking units. This is achieved by promoting *cis–trans* back reaction under the blue light (455 nm) illumination.

Recently, our group demonstrated that the exposure of an azobenzene containing chiral LCN to a combination of UV (365 nm) and blue light (455 nm) shows a large amplification of surface deformation when compared with the use of UV light alone.^31^ This was explained by the simultaneous excitation of both the *trans* and the *cis* azobenzene moieties thus enhancing the oscillatory effects and continuous dynamic straining of the network chains. To demonstrate that network dynamics also plays a role in repositioning of the glass transition temperature we recorded the storage modulus under similar exposure conditions.

**Fig. 3:**
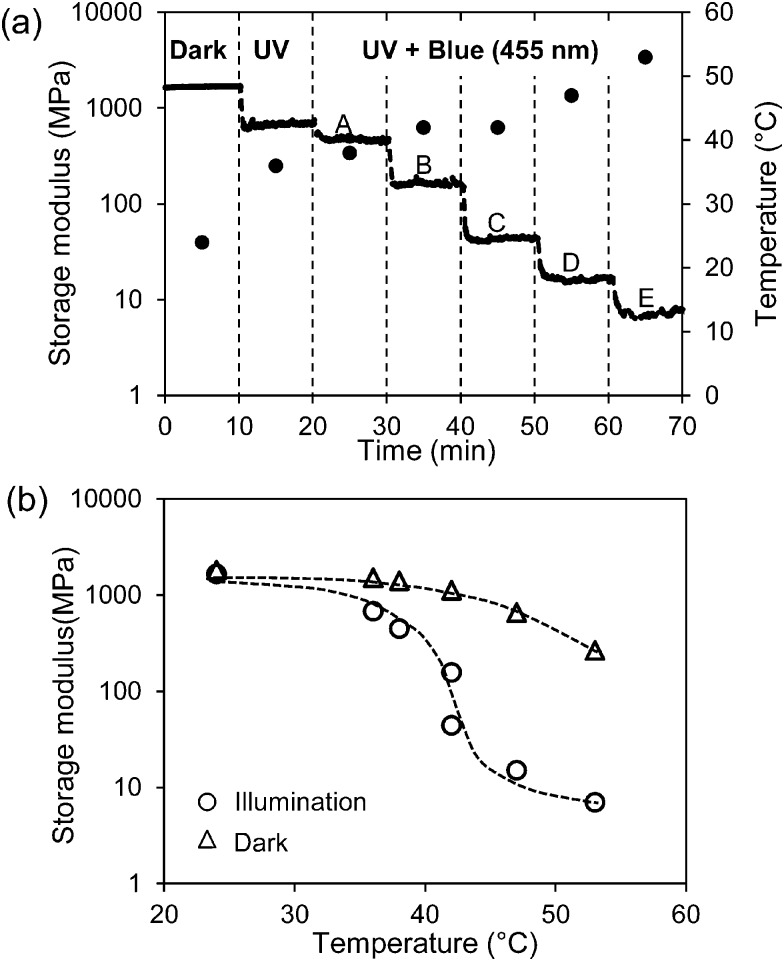
Change of the storage modulus of the low-crosslinked sample under various exposure conditions. (a) Storage modulus (solid line) and temperature () in the dark and only UV (365 nm) light exposure at the intensity of 300 mW cm^–2^. Regions A–E are illumination conditions with a continuous 365 nm UV light at 300 mW cm^–2^ and a stepwise increase of the intensity of blue light (455 nm) at 4.8, 18, 48, 99, 198 mW cm^–2^, respectively. (b) Comparison of the storage modulus at the same temperature with (○) and without (Δ) exposure (data derived from Fig. 1). Illumination conditions correspond to (a).

In Fig. 3(a) the course of the modulus is presented when stepwise more blue LED light (455 nm) is added to continuous exposure to 300 mW cm^–2^ UV light (365 nm). It shows that despite the fact that the back-reaction from *cis* to *trans* azobenzene is promoted the modulus keeps decreasing until finally the rubbery plateau is reached at around 10 MPa.

Increasing the intensity of blue light cannot prevent the temperature increase as shown in Fig. 3(a). The temperature increase on itself would also lead to a decrease in the modulus. This decrease can be derived from Fig. 1 and is also displayed in Fig. 3(b). It is obvious that the large decrease in the modulus under the combined exposure to UV plus blue light cannot be explained by the temperature alone and that additional softening effects are stimulated by blue light (more information on temperature and modulus changes under various illumination conditions are given in Fig. S1 and S2, respectively (ESI†)). Switching off the blue LED directly recovers the modulus of the LCN at its value with UV light alone. By further switching off the UV source the modulus goes back to its initial value before irradiation as presented in Fig. 1.

It should be noted that during all exposure experiments the liquid crystal order is maintained. In the dark a broad transition to an isotropic state can be observed by polarization microscopy at around 180 °C. Under the exposure conditions presented in Fig. 3 with the highest combination of UV and blue light intensities this transition is reduced to around 160 °C, still well above the experimental temperatures.

**Fig. 4:**
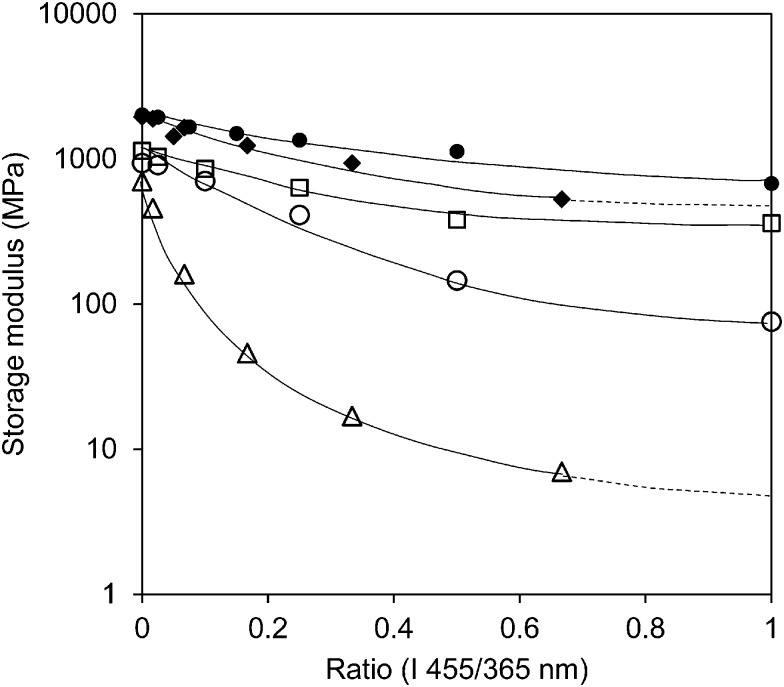
Storage modulus of the low and high crosslink density LCNs under various irradiation conditions. The open data points refer to low-crosslinked LCNs with a UV (365 nm) intensity of 100 mW cm^–2^ (), 200 mW cm^–2^ (○) and 300 mW cm^–2^ (Δ), respectively, in which the intensity of blue light (455 nm) has been increased. The close data points correspond to the high-crosslinked LCNs with UV light intensities of 200 mW cm^–2^ () and 300 mW cm^–2^ (◆), respectively.

The storage modulus is also measured for different levels of UV intensities in combination with a stepwise increase of blue light intensity (Fig S2 (ESI†)) and for the high-crosslinked sample (Fig. S3 (ESI†)). Fig. 4 illustrates that the intensity of the light sources (UV and blue light) has a large influence on the modulus. Although one should realize that at the lowest UV intensity the blue light intensity is correspondingly a factor of three lower as well. At high crosslink density the influence from the light sources is diminished. While the low-crosslinked samples show a modulus reduction of more than two orders of magnitude, the high-crosslinked sample is less than one order. Even at the highest intensity combination the reduction in modulus stays far above the rubbery plateau and seems to be the result of the temperature only (Fig. 1 and Fig. S3 (ESI†)).

The observations support the assumption that the oscillating strain of the network is the underlying mechanism for the light-induced plasticization. It also shows that it is a delicate interplay between the *cis*–*trans* modulation of the azobenzene and the length of the polymer main chains between the crosslinking junctions. Shortening the average length of these junctions with roughly a factor of 1.6 as calculated from the composition gives already a dramatic effect on the modulus reduction while the initial glassy modulus is only slightly different. In this respect it should also be considered that crosslinks partly consist of the oscillating azobenzene moieties.

**Fig. 5:**
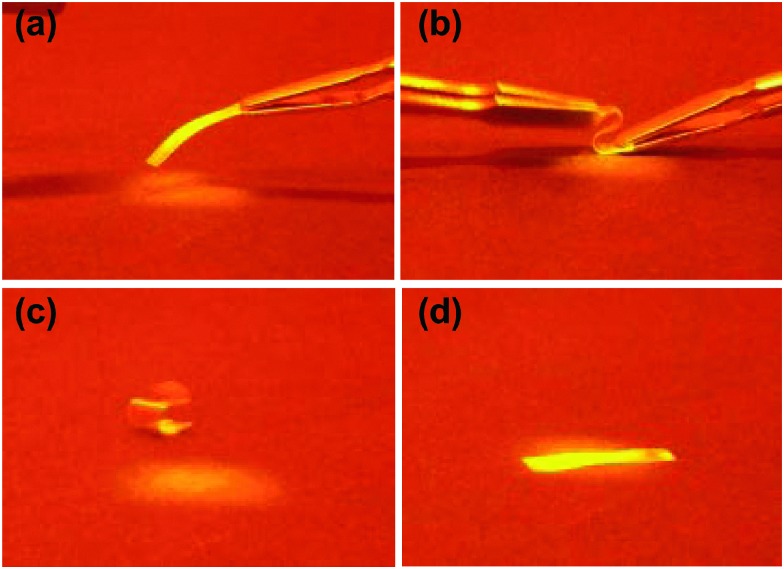
The shape memory effect in the liquid crystal polymer film: (a) before dual light treatment, (b) subjected to dual light illumination and deformed into a curled shape, (c) shape after the dual light treatment and (d) recovered the initial shape upon dual light exposure.

Reducing elastic moduli through UV illumination has been demonstrated to induce light controlled shape memory materials.^27,28^ Here we can use dual wavelength exposure by combining UV and blue light to tune the liquid crystal polymer network from glassy to the rubbery state as shown in the moduli data in Fig. 4. The light induced rubbery state of the LCNs gives deformation capabilities to the materials which can be frozen in by taking away the light source allowing the film transition to the glassy state and freezing in the new shape. Conventional shape memory polymers are deformed and fixed to a temporary shape, and recover to their original, permanent shape by changing the temperature below and above the glass transition temperature. Here we can use light to achieve a similar effect. The light induced shape memory of a low crosslinked liquid crystal network is illustrated in Fig. 5. Fig. 5(a) shows a free standing film of the polymer network before dual light illumination. The film is mechanically deformed in a curled shape in the presence of UV and visible light. The intensity ratio of the 455/365 nm light was 0.66 at 300 mW cm^–2^ UV light.

The deformed shape does not change over time in the ambient environment that shows permanent deformation of the network (Fig. 5(c)). The original shape of the polymer was obtained again upon exposure to dual 455/365 nm light (Fig. 5(d)). This process is recorded and found in the Video (ESI†).

## Experimental materials and methods

### Materials

Scheme 1 shows the mixture used to create smart coatings that can undergo frictional changes through exposure to UV light. Monomers 1 to 3 were obtained from Merck UK. Monomer 4 was obtained from BASF. Monomer 5 was custom-synthesized by Syncom (Groningen, the Netherlands). Polymer films were fabricated using a mixture containing 22 wt% monomer 1, 40.6 wt% monomer 2 and 31 wt% monomer 3, 3.4 wt% monomer 4, 2 wt% monomer 5, and 1 wt% photoinitiator 6 for the low-crosslinked composition and 32 wt% monomer 1, 35.6 wt% monomer 2 and 26 wt% monomer 3, 3.4 wt% monomer 4, 2 wt% monomer 5, and 1 wt% photoinitiator 6 for the high crosslink density composition. The constituents were mixed by dissolving in dichloromethane. DSC results show that the mixture has the chiral-nematic phase in the temperature range between 40 °C and 60 °C. At higher temperatures it becomes isotropic.

### Sample preparation

Glass substrates are cleaned by a 5 minute dip in acetone and 2-propanol subsequently while stirring, flushed with demi water followed by drying with nitrogen flow. A1051 (Sunever, Nissan Chemical, Japan) was used to obtain a planar alignment of the liquid crystal monomer mixture. It was spin coated on cleaned glass followed by baking. Gentle manual rubbing on a velvet cloth gave the substrate the desired alignment.

A cell having a thickness of 6 μm and provided with alignment layers was capillary filled with the monomer LC mixture and subsequently cured by UV exposure at 45 °C for 30 minutes with an intensity of 400 mW cm^–2^ using a mercury lamp (EXPR Omnicure S2000) equipped with a cut-off filter transmitting light >400 nm (Newport FSQ-GG400 filter) to prevent isomerization of the azobenzene group during polymerization. The samples were post-baked at 120 °C to ensure full cure of the acrylate monomers.

### Sample characterization

The cholesteric film was checked by polarized microscopy (Leica). The mechanical properties of the films are tested by DMTA (Q800 Dynamic Mechanical Analyzer from TA Instruments) at the frequency of 0.5. For this a sample of 14 × 4.2 × 0.06 mm dimensions was fixed at both ends in DMTA clamps and characterized in a strain-controlled mode. The measurements of photomechanical properties are carried out under continuous light illumination. LED lamps (Thorlab, M365L2 and M455L3) are used to provide monochromatic light. Temperature is monitored in a contact way using a thermal couple (Fluke, 80PK-1 K-Type Bead Thermocouple) and in a contactless way using an infrared camera (Fluke Ti-32).

## Conclusion

In this study we have demonstrated that the mechanical properties of a crosslinked liquid crystal network can be adjusted by light. A small amount of a copolymerized azobenzene crosslinker is capable of reducing the elastic modulus when exposed to UV light (365 nm). The effect is enhanced when blue light (455 nm) is added thus simultaneously stimulating the forward and backward *trans*-to-*cis* photoisomerization reaction, indicating that network dynamics play an important role in this process. At an optimized crosslinker content the polymer can be switched from its glassy state through the glass transition to the rubbery state. This phenomenon can be successfully utilized to make a memory material that switches between two geometrical shapes by light rather than by temperature.

## References

[cit1] Klajn R. (2014). Chem. Soc. Rev..

[cit2] Cheng Z., Wang T., Li X., Zhang Y., Yu H. (2015). ACS Appl. Mater. Interfaces.

[cit3] Dupin D., Rosselgong J., Armes S. P., Routh A. F. (2007). Langmuir.

[cit4] de Haan L. T., Verjans J. M. N., Broer D. J., Bastiaansen C. W. M., Schenning A. P. H. J. (2014). J. Am. Chem. Soc..

[cit5] Zhang W. L., Choi H. J. (2014). Polymers.

[cit6] Samui A., Jayakumar S., Jayalakshmi C., Pandey K., Sivaraman P. (2007). Smart Mater. Struct..

[cit7] Thévenot J., Oliveira H., Sandre O., Lecommandoux S. (2013). Chem. Soc. Rev..

[cit8] Medeiros S., Santos A., Fessi H., Elaissari A. (2011). Int. J. Pharm..

[cit9] Schmidt A. M. (2006). Macromol. Rapid Commun..

[cit10] Dong L.-c., Hoffman A. S. (1991). J. Controlled Release.

[cit11] Beharry A. A., Sadovski O., Woolley G. A. (2011). J. Am. Chem. Soc..

[cit12] Pace G., Ferri V., Grave C., Elbing M., von Hänisch C., Zharnikov M., Mayor M., Rampi M. A., Samorì P. (2007). Proc. Natl. Acad. Sci. U. S. A..

[cit13] Bandara H. D., Burdette S. C. (2012). Chem. Soc. Rev..

[cit14] Yu H. (2014). J. Mater. Chem. C.

[cit15] White T. J., Broer D. J. (2015). Nat. Mater..

[cit16] Tang R., Liu Z., Xu D., Liu J., Yu L., Yu H. (2015). ACS Appl. Mater. Interfaces.

[cit17] van Oosten C. L., Corbett D., Davies D., Warner M., Bastiaansen C. W., Broer D. J. (2008). Macromolecules.

[cit18] White T. J., Serak S. V., Tabiryan N. V., Vaia R. A., Bunning T. J. (2009). J. Mater. Chem..

[cit19] Liu Z., Tang R., Xu D., Liu J., Yu H. (2015). Macromol. Rapid Commun..

[cit20] Ware T. H., McConney M. E., Wie J. J., Tondiglia V. P., White T. J. (2015). Science.

[cit21] Liu D., Bastiaansen C. W. M., den Toonder J. M. J., Broer D. J. (2012). Macromolecules.

[cit22] Harrison J. M., Goldbaum D., Corkery T. C., Barrett C. J., Chromik R. R. (2015). J. Mater. Chem. C.

[cit23] Fang G., Maclennan J., Yi Y., Glaser M., Farrow M., Korblova E., Walba D., Furtak T., Clark N. (2013). Nat. Commun..

[cit24] Hurduc N., Donose B. C., Macovei A., Paius C., Ibanescu C., Scutaru D., Hamel M., Branza-Nichita N., Rocha L. (2014). Soft Matter.

[cit25] Vapaavuori J., Laventure A., Bazuin C. G., Lebel O., Pellerin C. (2015). J. Am. Chem. Soc..

[cit26] Petr M., Helgeson M. E., Soulages J., McKinley G. H., Hammond P. T. (2013). Polymer.

[cit27] Lee K. M., Koerner H., Vaia R. A., Bunninga T. J., White T. J. (2011). Soft Matter.

[cit28] White T. J. (2012). J. Polym. Sci., Part B: Polym. Phys..

[cit29] Lee K. M., White T. J. (2012). Macromolecules.

[cit30] Vapaavuori J., Goulet-Hanssens A., Heikkinen I. T. S., Barrett C. J., Priimagi A. (2014). Chem. Mater..

[cit31] Liu D., Broer D. J. (2015). Nat. Commun..

[cit32] Liu D., Bastiaansen C. W., den Toonder J. M., Broer D. J. (2012). Angew. Chem., Int. Ed..

[cit33] Yu Y., Nakano M., Shishido A., Shiono T., Ikeda T. (2004). Chem. Mater..

[cit34] Hikmet R. A. M., Broer D. J. (1991). Polymer.

[cit35] Harris K. D., Cuypers R., Scheibe P., van Oosten C. L., Bastiaansen C. W. M., Lub J., Broer D. J. (2005). J. Mater. Chem..

