# Supplementary material for: Regulating the modulus of a chiral liquid crystal polymer network by light
Source: Soft Matter. 2016 Feb 15;12(13):3196–201. doi: 10.1039/c6sm00114a (PMC5050537; doi:10.1039/c6sm00114a)
Supplement: Supplementary file 1 [file SM-012-C6SM00114A-s001.pdf]

Electronic Supplementary Material (ESI) for Soft Matter.
This journal is © The Royal Society of Chemistry 2016

Supplementary Information: **Regulating the Modulus in Chiral Liquid Crystal Polymer Network by Light**

Kamlesh Kumar,[a] Albertus P. H. J Schenning,[a, b] Dirk J. Broer[a,b] and Danqing Liu*[a,b]

[a.] Laboratory of Functional Organic Materials & Devices (SFD), Department of Chemical Engineering &Chemistry, Eindhoven University of Technology, De Rondom 70, 5612 AP, Eindhoven, The Netherlands

[b.] Institute for Complex Molecular Systems (ICMS), Eindhoven University of Technology, De Rondom 70, 5612 AP, Eindhoven, The Netherlands

(Dated: January 14, 2016)

Temperature changes under the light illumination

The temperature of the sample is examed by a thermal couple (Fluke, 80PK-1 K-Type Bead Thermocouple) and an infrared camera (Fluke Ti-32). The absorption of azobenzene molecueles cause the temperature to rise when exposed to UV light (365 nm). Adding blue light (455 nm) during continuous UV exposure results in a steady temperature increase.

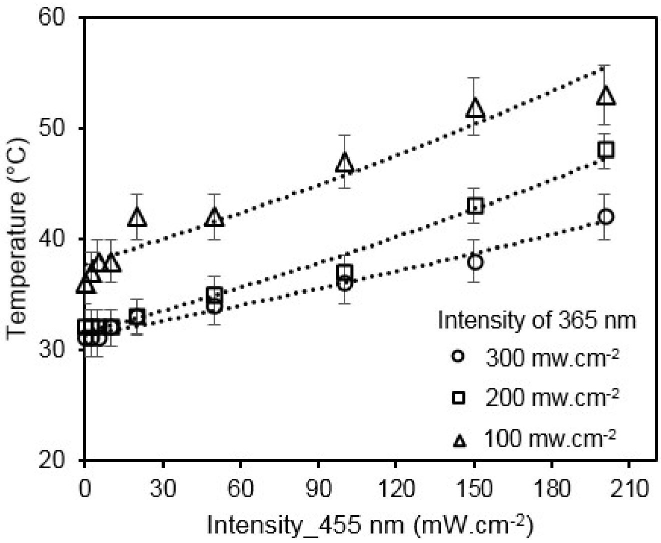

Fig. S1 Recorded temperature of the low crosslinked film under various illumination conditions. Continuous UV light at 300 mW.cm^{-2} (Δ), 200 mW.cm^{-2} (□), and 100 mW.cm^{-2} (O) are blended with increasing blue light (455 nm) intensities of 0, 2.5, 5, 10, 20, 50, 100, 150, 200 mW.cm^{-2}, respectively.

Modulus changes under different UV light intensities

In Fig. 3 in the main text, we present the modulus change of the low crosslink density sample under a blend of continuous UV (365 nm) light at the intensity of 300 mW.cm^{-2} and varied blue light (455 nm) intensities. Fig. S2 shows the effect of the intensity of 365 nm light by varing it at 100 mW.cm^{-2} and 200 mW.cm^{-2} while additional 455 nm light with different intensities are included.

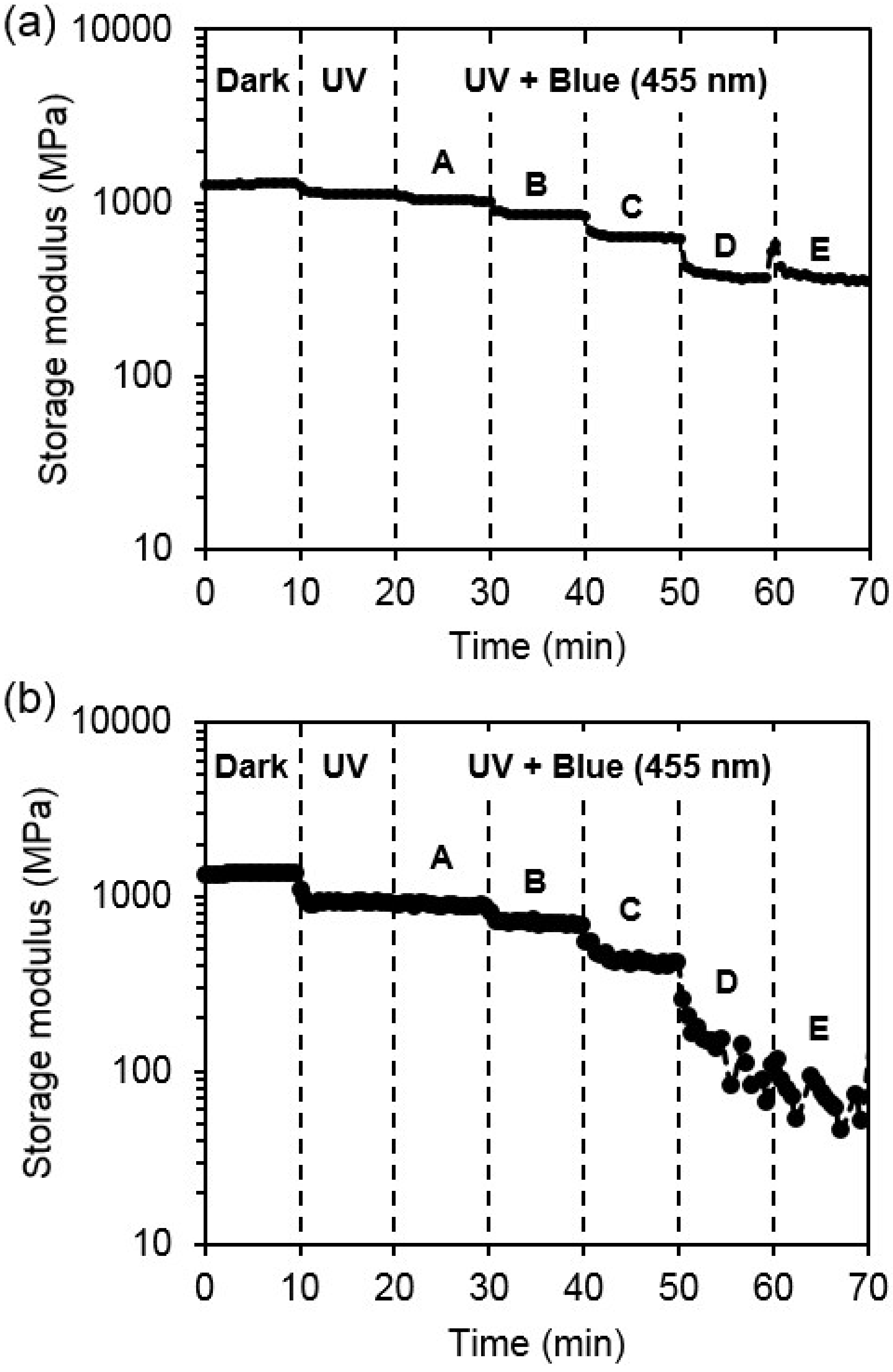

Fig. S2 Decrease of the storage modulus under the exposure. Mixed UV and blue light is used. UV intensity is kept at (a) 100 mW.cm^{-2} and (b) 200 mW.cm^{-2}. At a constant UV intensity extra blue light with the intensity of 5, 20, 50, 100, 200 mW.cm^{-2} are added as indicated in the region A-E, respectively.

Modulus changes of the high crosslinks sample

The effect of the crsslink density is investigated. A higher crosslinked sample with 37.4 w% of the crosslinker (compared with 27.4 w% of the low crosslinked sample) is subjected to the same experimental conditions as described in Fig. 3 (main text) and Fig. S2 in ESI. The storage modulus is measured in the dark, under UV (365 nm) illumination and at a constant UV intensity in combination with a stepwise increase of blue (455 nm) light.

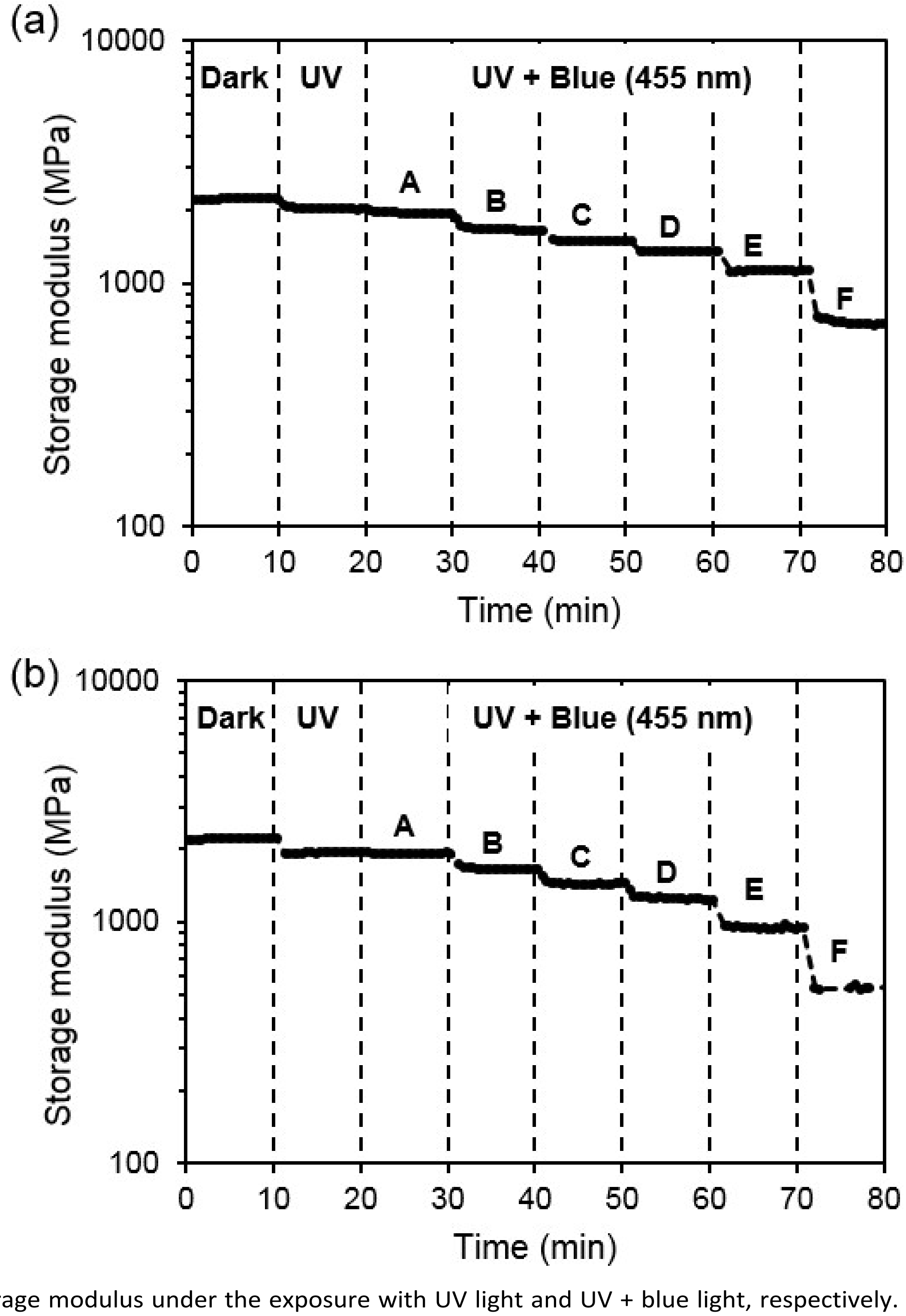

Fig. S3 Storage modulus under the exposure with UV light and UV + blue light, respectively. UV intensity is kept at (a) 200 mW.cm^{-2}, and (b) 300 mW.cm^{-2}. In region A-E (both (a) and (b)) the intensity of blue light is 5, 15, 30, 50, 100, 200 mW.cm^{-2}, respectively.
